# NorthBEAT: exploring the service needs of youth experiencing early psychosis in Northern Ontario

**DOI:** 10.3389/frhs.2023.1163452

**Published:** 2023-10-31

**Authors:** Chiachen Cheng, Shevaun Nadin, Hafsa Bohonis, Mae Katt, Carolyn S. Dewa

**Affiliations:** ^1^ Addictions and Mental Health, St. Joseph’s Care Group, Thunder Bay, ON, Canada; ^2^ Centre for Rural and Northern Health Research, Lakehead University, Thunder Bay, ON, Canada; ^3^ Clinical Sciences Division, Northern Ontario School of Medicine University, Thunder Bay, ON, Canada; ^4^ Dr. Gilles Arcand Centre for Health Equity, Northern Ontario School of Medicine University, Thunder Bay, ON, Canada; ^5^ Department of Psychiatry and Behavioral Sciences, University of California, Davis, Davis, CA, United States

**Keywords:** early psychosis intervention, youth mental health, rural/northern health services, underserved and unserved populations, barriers & facilitative factors early psychosis intervention

## Abstract

**Introduction:**

Early Psychosis Intervention (EPI) is critical for best outcomes. Among 369 diseases, psychosis is among those causing the greatest disability. Evidence-based interventions for youth in early stages of psychosis (EPI programs) have prevented chronic disability. Yet, EPI is frequently inaccessible for youth living in rural communities. Moreover, Indigenous youth often face more precipitous situations given inadequate staffing, and culturally unsafe care. The NorthBEAT (Barriers to Early Assessment and Treatment) project sought to understand the service needs of youth with psychosis in Northern Ontario. The goals were: (1) to describe the mental health of a subset of adolescents receiving EPI care; (2) examine Indigenous youth as a significant and vulnerable population; (3) to understand the barriers and facilitators for Indigenous and non-Indigenous youth receiving EPI.

**Methods:**

Mixed methods (structured and narrative interviews) included: psychometric scales interviews with youth, and narrative interviews with youth, their family, and service providers Data validation workshops were held with participants.

**Results:**

Structured interviews with 26 youth (*M* = 17 years) found the participants functioning moderately well with duration of untreated psychosis ranging from 1 to 96 months (*M* = 26 months). No significant differences were found in functioning or duration of psychosis between Indigenous and non-Indigenous youth. Narrative interviews were conducted with 18 youth, 11 family members, and 14 service providers. Identified barriers were a lack of knowledge about psychosis among service providers, a disconnected system leading to delays in treatment, help not wanted by youth, expansive geographical context. Service needs were: finding the right point of access, support for families, pre-crisis intervention, reduced stigma for youth and their families, and an EPI approach to care.

**Discussion:**

Rural and northern youth face similar barriers to accessing EPI as urban youth. However, northern youth face additional unique challenges due to expansive geographical context, limited resources and lack of knowledge about services.

## 1. Introduction

### 1.1. Psychosis and early psychosis intervention (EPI)

Psychosis, a symptom caused by serious mental illness such as schizophrenia, typically onsets in adolescence and can devastate youth and their families. Adolescents with psychosis can experience arrested development of skills which are necessary for achieving independent living, adult relationships, and career development (1). Amongst the 369 diseases and injuries measured by the Global Burden of Disease study, the disability weight of an individual experiencing an episode of psychosis is the highest, indicating a severe health state which can increase the risk of other outcomes such as suicide (2). Suicidal ideation ranges up to as high as 40% for people with psychosis, with the highest risk of suicide occurring in the first year of contact with health services (3, 4).

For best outcomes, psychosis requires early intervention and minimal duration of untreated illness. Early psychosis intervention (EPI) is an evidence-based intervention to treat young people in the early stages of psychotic illness, which is critical for ensuring improved outcomes in youth (5–8). Essential elements of EPI include early detection and identification, rapid access and assessment, intensive case management, family education, psychological treatments and low dose antipsychotic initiation; these elements have been reflected in multiple guidelines and standards (9–12). EPI is linked to lower all-cause mortality and reduced emergency department presentation (13) and improved outcomes (e.g., education and employment) that can be sustained post treatment (4, 14) One of the key features of the success of EPI is reducing the duration of untreated psychosis (4, 15, 16), also referred to as the DUP. EPI improves access to mental health services, and timely treatment reduces the long-term impact of psychosis.

### 1.2. Mental health needs in rural and remote communities

Although significant improvements have been made, Canada's mental health services fall short of meeting the needs of youth experiencing mental health disorders, especially for those living in rural communities (17). Adolescents living in rural communities tend to experience greater rates of mental illness, substance use, and suicide (18–20). This is exacerbated by factors such as increased social isolation, lower socioeconomic status, less educational opportunity, and fragmented mental health services (21). To date, the majority of the literature on the effectiveness of EPI programs has focused on urban areas. A recent review showed there is emerging evidence that EPI services in rural areas show similar positive patient outcomes compared to their urban counterparts, although the evidence base is limited in design and scope (22).

Indigenous people in Canada tend to experience poorer mental health (23), which stems partially from factors such as poorer social determinants of health and intergenerational trauma from residential schools (24). Mental health services in Indigenous communities are often inadequate due to challenges such as inadequate staffing, programs that do not provide culturally safe care, and insufficient funding (25).

### 1.3. Study context—Northern Ontario

Of Canada's 10 provinces, Ontario is the most populous and second-largest by area. While Northern Ontario has only 6% of the province's population, it has 80% of Ontario's landmass; it has an expansive geography with over five times the Indigenous population (15%) compared to rest of Ontario (2.8%) (26). Although Northern Ontario has 6% of the province's population, only 2 out of 50 provincial EPI programs service the region. Given the disparity in socioeconomic factors and access to mental health services between Indigenous and non-Indigenous youth in Northern Ontario, coupled with service providers' anecdotal experience that there is a difference in the severity of illness in these two groups, we sought to better understand this context. This would enable EPI services to better meet the youth needs, including unique presentations, experiences and care pathways.

### 1.4. NorthBEAT (barriers to early assessment and treatment)

The NorthBEAT research project sought to understand the service needs of youth with psychosis and their caregivers, with particular focus on marginalized groups such as Indigenous youth and youth living in northern and remote areas. The research question was: what are the perceived service needs of Indigenous and non-Indigenous youth in Northern Ontario who experience first episode psychosis? The objectives of this project were to (1) understand how youth in Northern Ontario experience first episode psychosis and services for psychosis; (2) to describe the mental health of a subset of adolescents receiving mental health care; (3) to specifically examine Indigenous youth as a significant and vulnerable population in Northern Ontario, and to engage Indigenous youth in a discussion about their service and access to mental health service needs; (4) to understand what are the barriers to and facilitators for Indigenous and non-Indigenous youth receiving appropriate early psychosis intervention.

## 2. Methods

This was a mixed-methods study that consisted of (i) structured quantitative interviews with youth who had accessed EPI services; and (ii) semi-structured qualitative narrative interviews with youth, caregivers, and key informant service providers. The goal of the structured interviews portion of this study was to provide a descriptive overview of the functional status of a sample of youth accessing services for psychosis in Northern Ontario. Narrative interviews were conducted to explore the experiences of youth and caregivers in accessing these services, as well as the perspectives of service providers.

Institutional ethics approval was received from St. Joseph's Care Group (SJCG), Lakehead University, and the Centre for Addictions and Mental Health. Full ethics approval was also received from the project sites (based at hospitals) which had research ethics boards (Health Sciences North, North Bay Regional Health Centre, Sault Area Hospital, and SJCG). Delegated approval was obtained from program managers at the project sites which did not have research ethics boards.

To meaningfully engage Indigenous communities, co-author M. Katt, who is Indigenous from the Temagami First Nation in Northern Ontario, with extensive research with Indigenous communities (27, 28) was involved with every aspect of this project, starting with developing the research question. Researchers met with leaders in Indigenous communities (e.g., Nishnawbe-Aski Nation and Wikwemikong First Nation regarding the feasibility of participation on their land). To strengthen the participatory nature of the study, the NorthBEAT project also had a five member advisory group. The purpose of the advisory group was to help guide the project, advise about how to engage participants, provide diverse perspectives and provide feedback about appropriate forums for knowledge translation activities. Members of the advisory group were: a younger adult who previously was a service-user, a family caregiver who is also Indigenous, social worker from one of the northwest project sites, staff members from Indigenous organizations such as the provincial Indigenous women's association, and nurse from one of the northeast project sites. Data collection occurred from 2013 to 2015. After data analysis was completed in 2015, knowledge translation and data validation workshops were held with project participants.

### 2.1. Participants

Participants were recruited from early intervention in psychosis programs based in Northern Ontario. Participants were *youth* who were receiving mental health services for psychosis, *family* caregivers of youth in mental health services, and youth mental health *service providers*. Sampling was purposive, and differed slightly by participant group and method of interview. The sampling strategies are described for each method below.

### 2.2. Structured quantitative interviews

Structured quantitative assessments were used to obtain a functional snapshot of a subset of youth who were receiving services of psychosis.

A purposive (criterion-based) sample of youth were recruited from ten project sites across Northeastern and Northwestern Ontario: First Place at Canadian Mental Health Association (Thunder Bay), St Joseph's Care Group (Thunder Bay), Step Program at Sault Area Hospital (Sault Ste. Marie), Mental Health & Addictions Program at Health Sciences North (Sudbury), Community Mental Health Service (Muskoka/Parry Sound), Mental Health Clinic at North Bay Regional Health Centre (North Bay), Minto Counselling Services (Iroquois Falls), Payukotayno Family Services (Moosonee), Weeneebayko Health Authority (James/Hudson Bay Lowlands) and Canadian Mental Health Association (Cochrane/Timiskaming). These sites were selected because they were the Northern Ontario early psychosis intervention programs based in the community. Clinicians at the project sites informed their eligible clients of the study, and connected them to the researchers for enrollment in the study. Consent was obtained with youth using an innovative process (29), and potential participants were given the opportunity to decline to participate. Eligible youth were either (i) 18 years of age or younger; or (ii) had accessed the services as an 18-year-old or younger in the past two years (i.e., the maximum participant age was 20). In addition, all youth participants were able to understand and be interviewed in English, and were capable of providing informed consent.

The interviews were conducted by phone, and included an assessment of several domains. The domains measured were demographics, duration of untreated psychosis (Nottingham Onset Schedule) (30), observer-rated mental health [Positive and Negative Symptom Scale (PANSS) (31, 32) and Global Assessment of Functioning (GAF) (33)], client-related mental health [Brief Symptom Inventory (34)], and recovery/psychosocial function (Recovery Assessment Scale) (35).

Demographic data that were collected included sex, age, racial background, marital and children status, level of education and income, housing, area/region where they live. The racial background was used to create an Indigenous/non-Indigenous variable. This dichomotous variable indicated whether the respondent self-identified as a member of an Indigenous community.

These quantitative measures were selected for their psychometric properties, brevity (outside of the PANSS, the other scales take approximately one hour to administer in total) and commonality with other EPI studies. The commonality helps with comparing to early intervention service models used elsewhere and the existing literature. Also, we had previous data from the Matryoshka Project (36, 37) about the needs and mental health status of clients experiencing psychosis in six Ontario urban and rural EPI programs, however the participants were 18 years and older. The measures selected for the current project were valid for 18 years and younger. Other standard tools used in child and adolescent mental health services that measure behaviour, functioning and presence of other mental disorders such as Brief Child and Family Phone Interview (38) and Child and Adolescent Functional Assessment (39).

The structured interviews also used measures of psychosis dimensions. The Nottingham Onset Schedule (NOS) measures the duration of psychosis by measuring the time between the first observed change in mental state/behaviour and the onset of psychosis symptoms (30). While the inter-rater reliability was 65%–90%, and test-retest reliability was 80%–100% (depending on the dimension being measured for the NOS), it is widely acknowledged in the field that measuring DUP has its challenges, including recall bias (40). The PANSS is a 30-item instrument which measures the severity of schizophrenia symptoms across three dimensions: positive (7 items), negative (7 items), and general psychopathology (16 items) (31, 32). Each item is scored on a 7-point Likert scale, and once summated scores range from 7 to 49 across the positive and negative scales and 16–112 across the general psychopathology scale. The Global Assessment of Functioning scale was used as part of Diagnostic Statistical Manual of Mental Disorders IV- TR (33) to assess the severity of mental illness symptoms impact on daily functioning. The Brief Symptom Inventory uses a 5-point Likert scale to assess 53 items across nine dimensions: somatization, obsessive-compulsion, interpersonal sensitivity, depression, anxiety, hostility, phobic anxiety, paranoid ideation, psychotism, as well as the Global Severity Index, Positive Symptom Distress Index, and Positive Symptom total (34). In people recovering from severe mental illness, the 24-tiem Recovery Assessment Scale measures personal confidence and hope (9 items), willingness to ask for help (3 items), goal and success orientation (5 items), reliance on others (4 items), and no domination by symptoms 3 items (35).

Using SPSS software, these data were analyzed to provide a descriptive snapshot of the demographic characteristics, mental health status, psychosis symptoms, psychosocial functioning, and duration of untreated psychosis in both groups. An independent two-samples *t*-test was done to explore whether there were significant differences between Indigenous and non-Indigenous participants in the domains assessed. Dependent variables were continuous, and independent variable was ethnicity (e.g., Indigenous, non-Indigenous). Clinical significance (i.e., “caseness”) as indicated by either a Brief Symptom Inventory (BSI) *T*-score equal to or greater than 63, or a *T*-score of equal to or greater than 63 on any two subscales (34).

### 2.3. Narrative qualitative interviews

Semi-structured narrative interviews were conducted to explore the care experiences and perceived service needs of northern youth who experience psychosis.

#### 2.3.1. Youth and family members

Sampling and recruitment strategies for the youth were consistent with the Structured Interviews (described above). After the structured interviews, youth participants were asked if they would be interested to be contacted at a later date for a narrative interview.

*Family members* or caregivers of youth who met the inclusion criteria were also eligible to participate. They did not need to be from the same family unit as the youth. All family participants were able to understand and be interviewed in English, and were capable of providing informed consent. Just like the youth, family members were informed about the study by the frontline clinicians at the project sites. Potential study participants were provided with a brief overview of the study, and those who expressed interest were connected with the NorthBEAT researchers. Researchers also reviewed the purpose of the study. At that point, prospective participants were able to decline to participate. Informed consent was obtained from those who were willing to participate.

#### 2.3.2. Service providers

**Figure 1:**
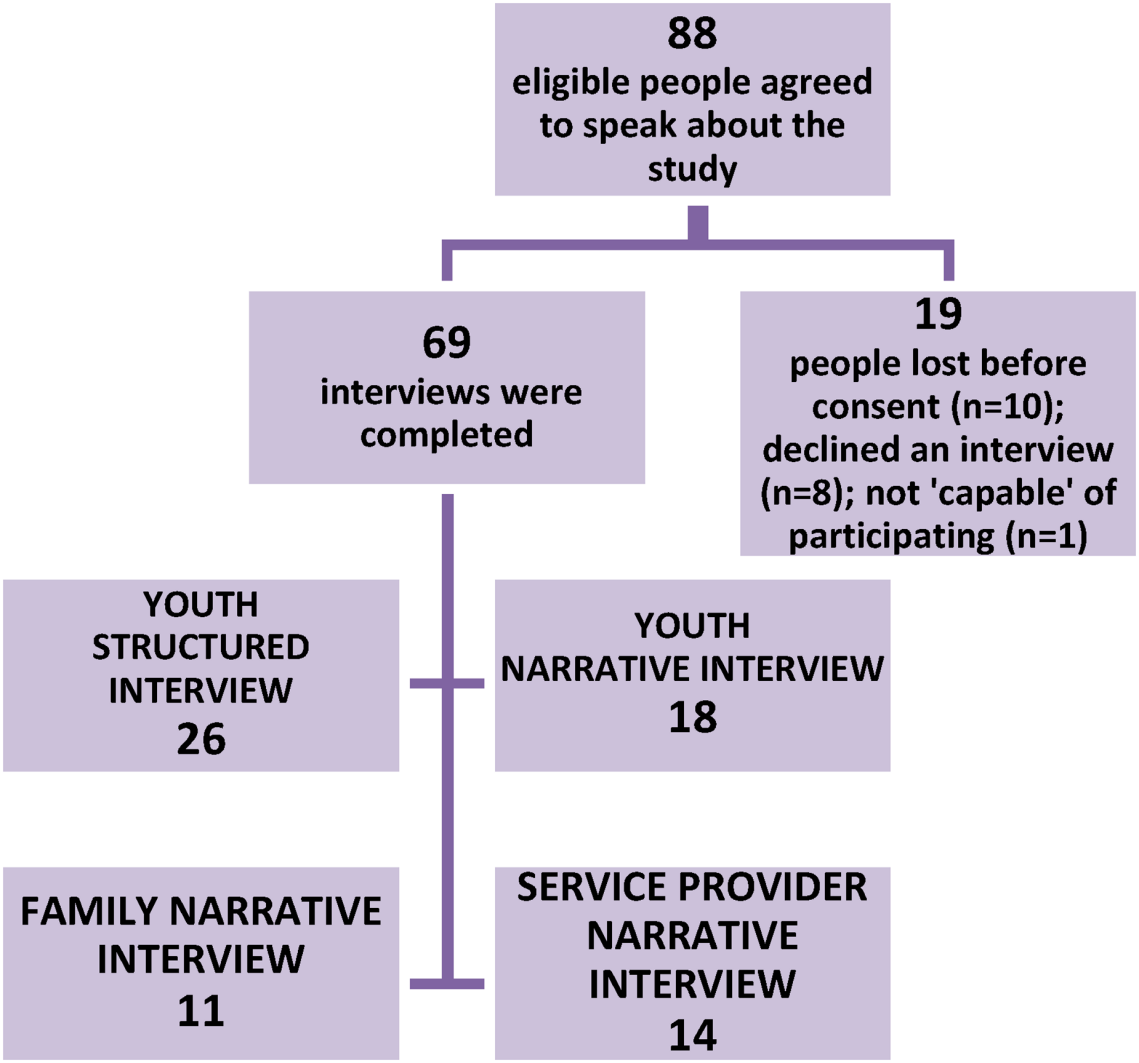
Recruitment flowchart and number interviewed.

Purposive (maximum variation) sampling was used to recruit key informant service providers who had either managed or provided direct mental health services to youth. The sample was curated to provide broad representation of the service needs of Indigenous and non-Indigenous youth across Northwestern and Northeastern Ontario (dimensions of variation were nature of work, geographic location, and knowledge of Indigenous and non-Indigenous youth mental health clients' needs). Researchers sent letters of invitation that described the study to key informants; interested service providers contacted research coordinator and were scheduled an interview after consent was obtained. See Figure 1 for a recruitment flowchart.

All interviews were conducted by phone. Interview questions probed youths' help-seeking experiences as well as participants' perceptions about what youth with psychosis need from health care. The interviews ranged in length from 9 to 33 min for youth (average 19 min), 18–47 min for family members (average 34 min), and 27–65 min for service providers (average 46 min). All interviews were audio recorded, and transcribed verbatim for analysis. Interviews were conducted until theoretical saturation was reached in each of the participant categories. Saturation was reached when the researchers felt that the interviews (across the sample groups) were not generating any new information.

Youth and family member participants received compensation of either a gift card or monetary compensation with a value of $40 CAD.

Transcripts were analyzed inductively, using a thematic networks approach (41). The principal investigator and research coordinator performed a detailed analysis of the interview transcripts, and worked with the project research assistant to develop a coding framework. NVIVO software was used to organize and code each transcript, which was then verified by the principal investigator.

### 2.4. Data validation workshops

After data analysis was completed, data validation and knowledge exchange workshops were held with project participants and stakeholders. The objectives of these sessions were to (1) validate the data analysis, (2) co-create arts-based products from the study data as a mechanism for knowledge translation, (3) work with stakeholders to determine possible knowledge translation audiences and venues, and (4) to seek permission to use the study findings at knowledge translation events.

In June 2015, separate workshops were held for youth participants, caregiver participants and service providers or other stakeholders. These participants had previously given consent to be contacted to discuss the study's results. Youth and caregiver sessions were held simultaneously in Thunder Bay and North Bay and linked by videoconferencing. The stakeholder meeting included all project investigators, collaborators, advisory group members, and representatives from each project site. All of these sessions were facilitated by a professional facilitator with experience leading data validation/knowledge translation workshops with vulnerable populations. During the workshops, participants were involved in a range of activities which were intended to validate preliminary findings. Ideas for how the research findings should be disseminated were discussed with all three groups, and examples of social media posts, campaign t-shirts and other knowledge exchange products were developed.

## 3. Results

### 3.1. Structured interviews

Of the 26 structured interviews which were conducted, 17 were from Northwestern Ontario and 9 were from Northeastern Ontario. Participants had a mean age of 17 years, and 54% were female. 61.5% were Caucasian, 27% Indigenous, and 11.5% were classified as “Other” ethnicity. 85% were enrolled in high school or post-secondary school, although 73% had experienced mental health related interruptions to their schooling. 58% were employed (paid or volunteer work) in the previous 12 months. 77% lived with family, and 46% paid rent. 99% of the participants had no physical illness.

The duration of their untreated psychosis (DUP) ranged from less than 1 month up to 96 months (mean = 26 months, median = 12, IQR = 36). Only 23% of participants had a duration of untreated psychosis of less than 3 months.

On the PANSS, the sample scored slightly below average on the positive scale (*M* = 39.65, SD = 8.54), and much below average on both the negative (*M* = 32.77, SD = 5.13) and general (*M* = 32.77, SD = 7.72) scales, indicating that they were experiencing fewer symptoms and general psychopathology than the normative sample of medicated patients with schizophrenia. 90% of participants scored 60 or higher on the GAF scale, indicating mild-moderate symptoms. Participants reported high levels of personal confidence and hope (*M* = 32.38, SD = 7.28), goal and success orientation (*M* = 19.65, SD = 3.54), and not dominated by psychosis symptoms (*M* = 9.42, SD = 3.58). On the BSI, half of the participants (*n* = 13) had BSI-T or *T*-scores equal to or greater than 63, indicating clinical significance.

**Figure 2:**
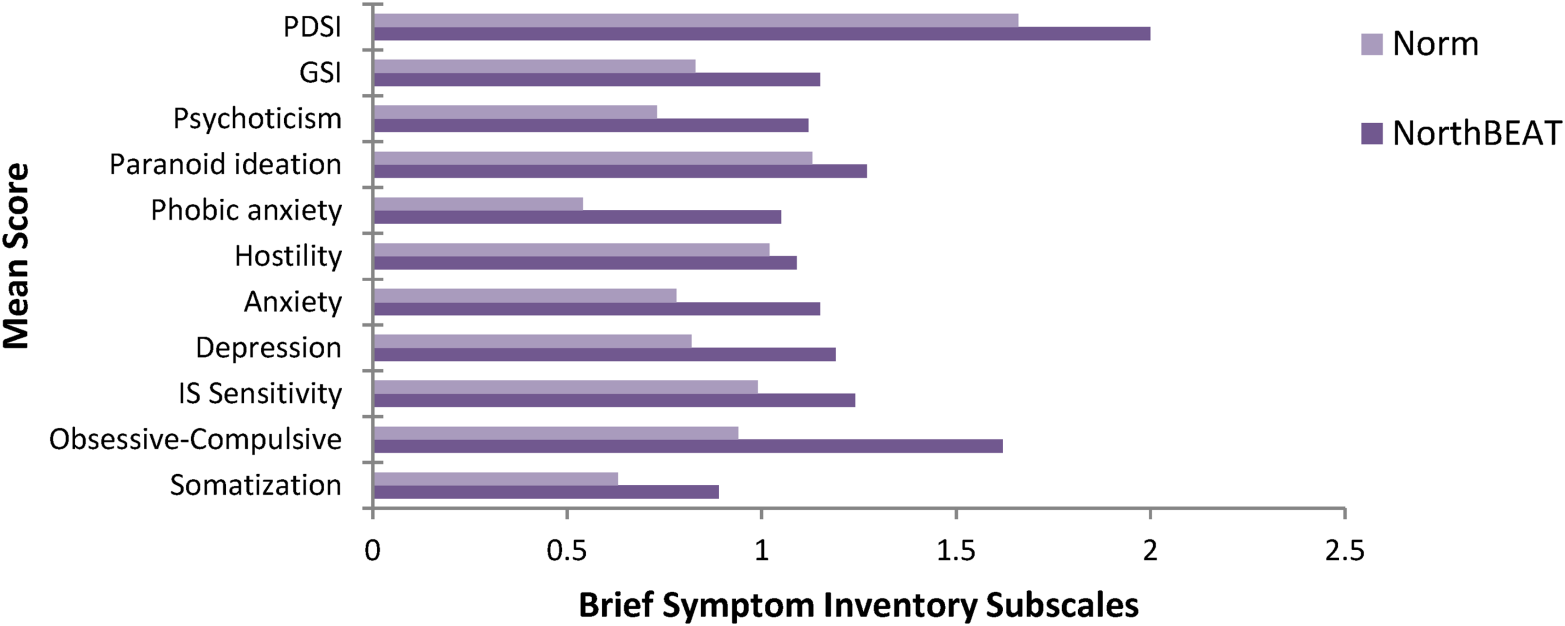
Mean psychological distress scores of NorthBEAT participants compared with published norms (means) from adolescent non-patients (34). IP, interpersonal; GSI, Global Severity Index; PDSI, Positive Symptom Distress Index.

See Figure 2 for a comparison of this sample with normative sample. There were no differences between Indigenous and non-Indigenous youth in duration of psychosis or any other indices (independent samples *t*-tests, *p* > .05).

### 3.2. Final framework from narrative interviews

A total of 43 narrative interviews were conducted. All 18 youth who completed the narrative interviews also participated in the structured interviews using measurement tools. Data were not collected about the number of youth—family/caregiver pairs due to the independent consent processes approved by the REBs. The service providers participants were from the 10 project sites (which were all EPI programs); data about whether the service provider and youth or family/caregiver were recruited from same sites was not collected.

**Figure 3:**
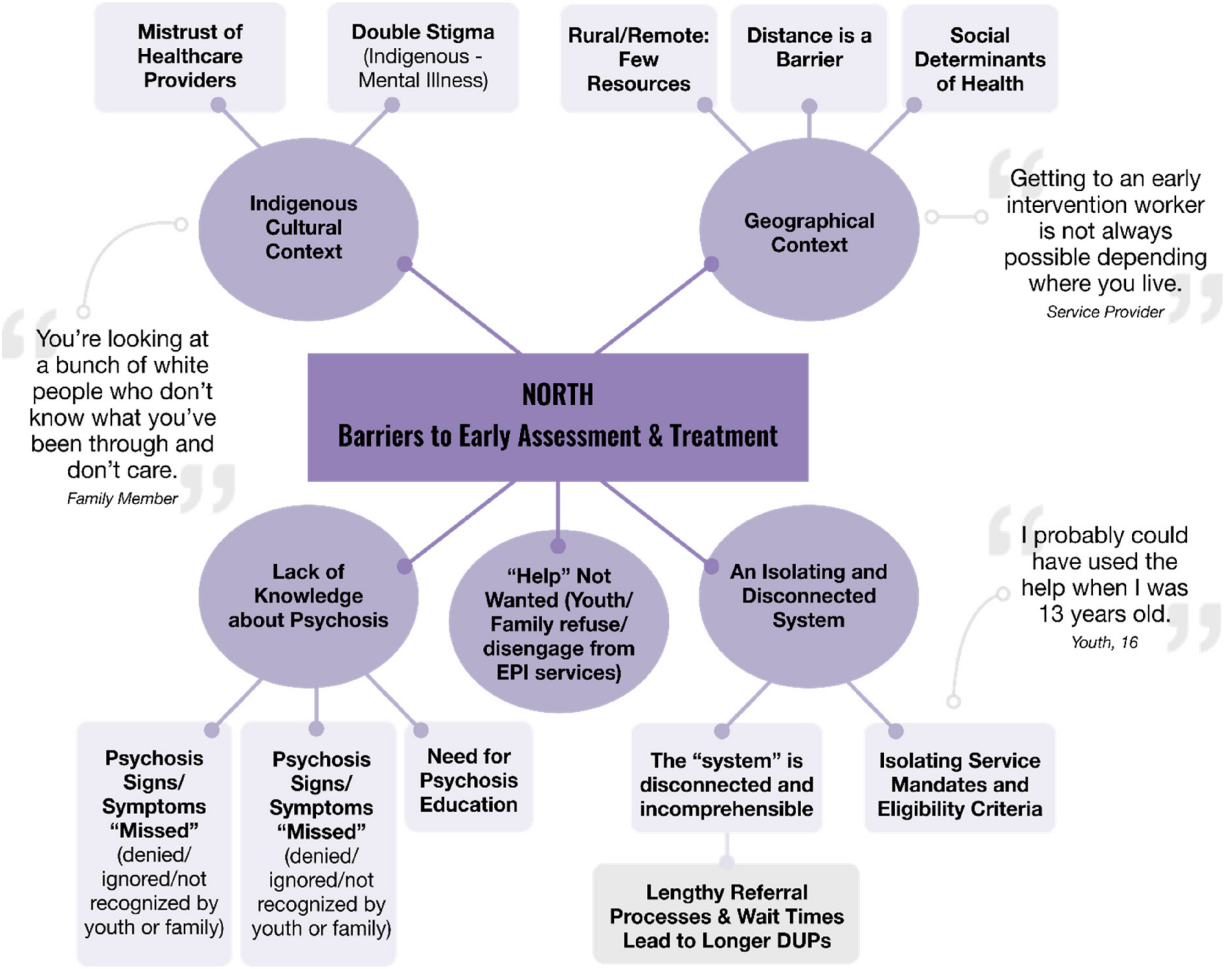
Barriers to early assessment and treatment.

**Figure 4:**
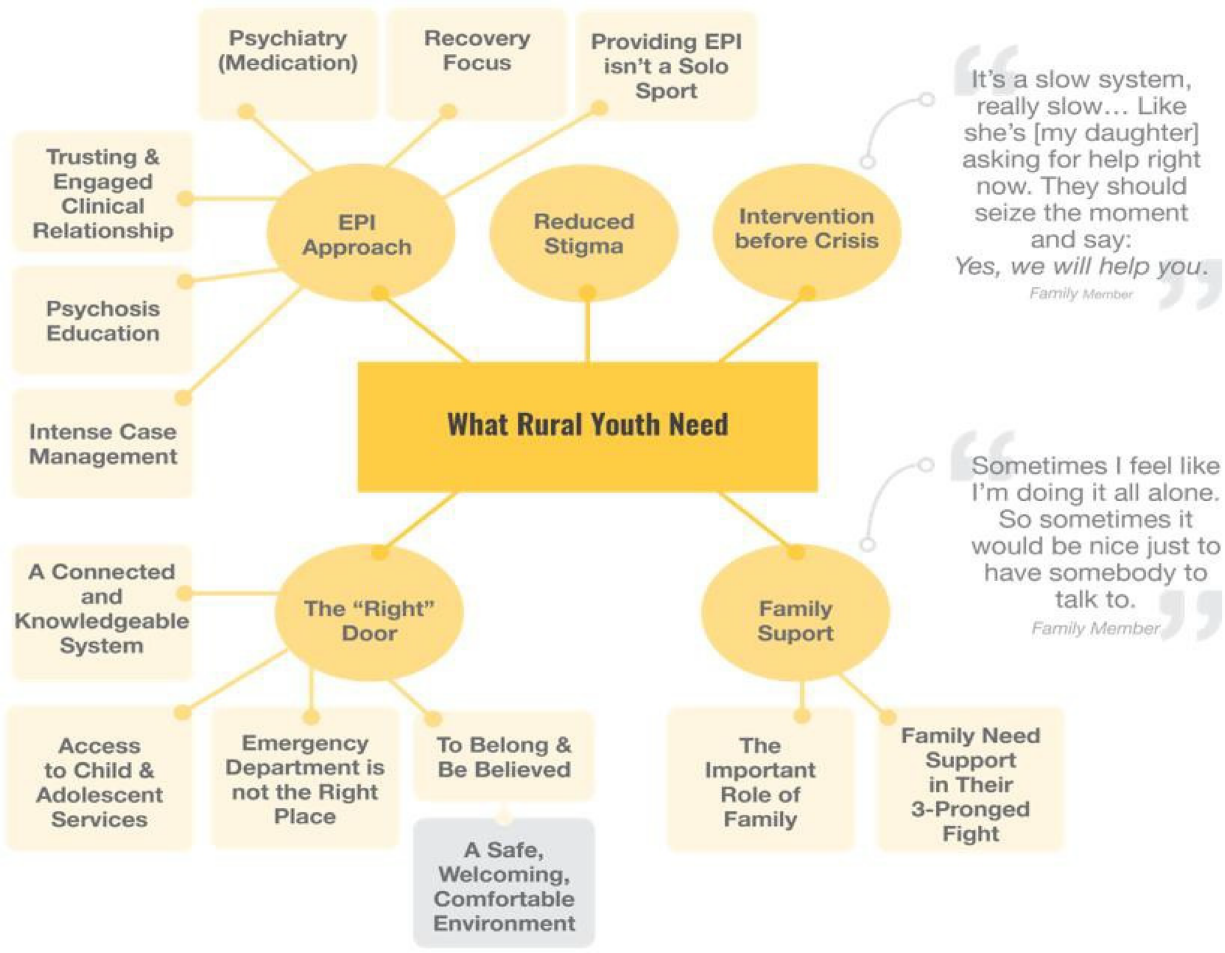
What rural youth need.

Analysis of the interviews resulted in two global themes: “North B.E.A.T. (Barriers to Early Assessment and Treatment)” and “What Youth Need”. These are presented using two thematic maps (see Figures 3, 4).

#### 3.2.1. Barriers to early assessment and treatment

Many youth and family caregivers experienced non-linear pathways to care. Their stories highlight important barriers that youth face in accessing EPI services. Five overarching themes and 12 subthemes were prominent under the global themes map: Barriers to Early Assessment and Treatment. (Figure 3).

##### 3.2.1.1. Lack of knowledge about psychosis

Participants explained that a lack of knowledge about psychosis was a significant barrier to early interventions. Even with family members who have above average knowledge about mental health issues, both youth and family participants described ignoring or not recognizing the early symptoms or signs of psychosis. A tendency to initially ignore psychosis contributed to delays in help seeking:

“Well, um, the first time it started like things started to like I noticed that some things was changing in him and they started like…first I kept it to myself like trying to ignore it, like make it go away, like not thinking about it or whatever.” (Family Member #1)

Youth and family members also turned to various service providers (e.g., counsellors, family physicians, children's mental health services, emergency departments) who either did not believe their stories, or did not recognize the signs and symptoms of psychosis. This was described as a very isolating experience, and a barrier that lead to longer duration of untreated psychosis for some youth:

“But I think if I had gotten the proper support when I was younger, instead of the doctor brushing it off, I think it would have helped me a lot more with like everything else in my life. Because I haven't been able to finish school or have a proper social life or anything like that because I never got the proper support at first.” (Youth #1)

These “missed opportunities” were very isolating for youth and families, and often meant that the youth became sicker, or more symptomatic before they received help. In situations where providers did recognize the psychosis and knew how to access EPI services, the pathway to care was more direct and treatment facilitated.

Participants often suggested education is needed at the various points of access so that non-specialist service providers can recognize early psychosis, know where to refer and appropriately intervene. Suggested focus for education included, teachers, guidance counsellors, community counselling centres, health care workers (e.g., work-place counselling services, telehealth, nursing stations in remote communities, family physicians, family health teams, emergency department staff), police officers or first responders, First Nations organizations and communities:

“It's about building capacity in the communities. I think people tend to brush it off. Oh, they’re talking a little bit funny.” (Service Provider #1)

The need to educate youth and families, and for more public awareness about psychosis symptoms and where to turn for help was a common theme. Suggested platforms included pamphlets, flyers, media blitz specific to northern context, presentations, commercials, quick little ads, social media so that the information about services and how to access is readily available in an easily understood format:

“Working with youth in general, that they have more information, and that they are better able to identify what could possibly be early symptoms of a first episode psychosis. So that people get identified earlier and referred to programs earlier, if they require more support in their home communities, that people have the resources and knowledge and the skills to provide those supports.” (Service Provider #2)

##### 3.2.1.2. Help not wanted

Families described frustration about not being able to intervene until a crisis arises. Before the crisis point, there were times that families sought out help but youth experiencing the psychosis declined. This *help not wanted* outcome was a barrier to accessing services. Families described the situation as a three-pronged fight that families engage in, fighting the independence of the youth, fighting the psychosis and navigating a complex system:

“The only barrier he faced was not knowing that he needed help…he didn't care if he got help.” (Family Member #1)

“It [the EPI program] only works as much as the client wants to give though. So, if I want to hide something, I could have easily, but I chose not to because I wanted to seek proper help. But, in that state of mind you can, you know, it would make you not want to reveal yourself.” (Youth #2)

##### 3.2.1.3. An isolating and disconnected system

System level barriers and youth resistance to accepting help led to a mental health system that is difficult to navigate for youth and their families. There was a disconnect between community care and acute care. Furthermore, mental health services are not well integrated into the health care system. This was frustrating and isolating for many of the youth and family members. Often, they were unaware about the EPI services available in their community until they experienced the convoluted, disconnected system and was referred there by a knowledgeable non-EPI service provider:

“It was just not knowing where to go…and how hard it was …you know…my husband is a [police] officer. And I work for one of the school boards. And so…we’re able to do these things. And then, there are families that don't have, maybe, quite the same skills. And if it was this hard for me, I have no idea how somebody, you know, that doesn't have the same skills would be able to help their child.” (Family Member #2)

Furthermore, service mandates and program eligibility criteria were significant barriers to early intervention. Age of client was a commonly described eligibility barrier. In many areas, pediatric mental health services were not available, or EPI programs were embedded in adult mental health programs (with client minimum age criteria of 16 years or 18 years old). Other barriers included exclusion criteria (e.g., bipolar diagnosis) or the requirement for a physician referral:

“I probably could have used the help when I was 13 years old, but it wasn't really available to me at that time just because of my age…if there were services that were like dedicated to helping young people, like even younger than like adolescent, then that might have helped me.” (Youth #3)

This youth participant went on to state that they received first intervention with acute admission at 19 years of age.

##### 3.2.1.4. Geographical context

Geographical context created barriers because in rural, small or remote communities, participants had limited access to the appropriate health care providers such as Adolescent Psychiatrists or very long wait lists to access services. Participants also explained that service needs outweighed resources, and capacity, and that provider burnout was often high. Due to overcapacity and lack of resources, some programs are not able to service their entire service catchment area and the solution of limiting caseload numbers, or limiting eligibility criteria further increased barriers. Other barriers included the vast distances participants had to travel to access appropriate services, or the impact of poverty and other social determinants of health which further increased barriers due lack of access to vehicle or transportation.

“In terms of looking at the geography of Northern Ontario, you have to travel so far to access anything. And so getting to an early intervention worker is not always possible depending where you live.” (Service Provider #3)

##### 3.2.1.5. Indigenous cultural context

Indigenous youth who experience psychosis in Northern Ontario may experience unique, additional barriers to early intervention compared to their non-Indigenous counterparts. These barriers included double stigma and mistrust of the healthcare system. Participants described the double stigma of having an Indigenous identity and a mental health problem. Participants who self-identified as Indigenous stated that they often felt mistrustful of the healthcare system because of previous negative experiences or because the care settings lacked cultural safety:

“It's just like mental health is stigmatizing, very stigmatizing, and then being Native is very stigmatizing. So you’re labeled right away, like you know, and it, it probably really sucks being a Native with a mental health disorder”. (Family Member #3)

Due to historical injustices, such as colonialization or Indigenous residential school system where Indigenous people experienced racism, Indigenous people may be reluctant to seek help:

“You’re looking at a bunch of white people that don't know what you’ve been through and don't care. So if you’re Native with mental health problems, that's what you see when you go to the hospital. You don't see other Natives there helping, or doctors, Natives who are doctors. You know, you see a whole bunch of white people who don't care.” (Family member #4)

Moreover, there may be a delay in recognizing signs and symptoms of psychosis because some of the communities are in crisis, dealing with the multiple inter-generational trauma from colonialization and the Indian residential school system (42).

#### 3.2.2. What youth need

The 5 overarching themes and 13 sub-themes captured in this second global theme map are the facilitators for early intervention; what youth who experience psychosis need in Northern Ontario (Figure 4).

##### 3.2.2.1. The “right” door

A well-connected and knowledgeable healthcare system would address the non-linear pathways to care. Participants expressed the importance that wherever youth and families seek help, they are able to find “the right door” to walk through. Furthermore, they hoped that every door was the “right door”. Families and youth described frustrations that the mental health system is not well integrated into the healthcare system, convoluted referral processes, and a need for better communication between community and acute care:

“It was like walking around like a blind man bluff, pin the tail on the donkey kind of thing. Whoever I talked to I tried to get as much information as possible. Whoever they referred me to, I went to. And everything, it takes like three or four months to get in.” (Family Member #5)

They also expressed the need for community and hospital-based services to be able to recognize psychosis, and know how to access the specialized EPI programs. In addition to EPI services, access to general adolescent mental health services was needed. Many noted the lack of pediatric psychiatrists and clinical psychologists, especially for youth living in more rural and remote regions. One youth explained “*because of my age, no one would take me. I was 14 (years) at the time”*. Another family member stated:

“It was so frustrating…Grade 9 it was, it was very clear he needed help and he wanted help and there was no where to go. Their school said there is nothing for anger management. And when we called Mental Health, Children's Mental Health, they said there's nothing; they have to wait until he's 17…It was more than just being 17, there was something else, there was also a waiting list 1½ years or something.” (Family Member #6)

When services made allowances for clients who were younger than typical eligibility criteria required; this was an important facilitator for earlier intervention.

Overall, participants spoke about less invasive settings as the right door. They strongly expressed that emergency departments and adult inpatient settings were not the appropriate places for youth experiencing psychosis. It was difficult to get the youth to accept help, and these settings discouraged ongoing treatment for the psychosis:

“I wish you didn't have to go through Emerg every single time. Because even waiting in there with him, we had to wait like two or three hours and then we had to sleep in Emerg overnight. And, by that time, he thought he was better because they gave him something to relax him and he didn't want to be admitted.” (Family Member #7)

The right door also included feeling a sense of belonging and clinicians believing their experiences were real. When reaching out for help, they need to have their concerns taken seriously. They also need a warm, welcoming and youth-friendly environment (these are foundational elements for EPI programs). They need providers at access points to know about early psychosis, and about EPI services in their community and facilitate care:

“I started experiencing it when I was like 13 but I didn’t get the proper help because they said, like the doctor that I was seeing, kind of just brushed it off” (Youth #4)

##### 3.2.2.2. Family support

Especially in psychosis in youth, families play a key caregiving and recovery role. The important role of family support is recognized in the themes that emerged. Often it is the families who initiate help-seeking, and families often provide supports to the youth so that they stay in services once they are referred. The family members spoke about needing to be tenacious in navigating the healthcare system, and providing support for their loved one. Having family support is an important facilitator. Moreover, supporting families in their three-pronged fight (i.e., fighting psychosis, fighting the system, fighting the independence of the youth) is crucial long-term:

“Well at first my foster mother was very unsupportive. She didn't really believe me when I explained to her that I was seeing things and hearing things, and it's, I don't really blame her because it's not really something you’d want to hear from your child, but (short pause) I, I believe if I received help from her sooner, things would have gone better with my doctor also”. (Youth #5)

##### 3.2.2.3. Intervention before crisis

Many participants described their experience of reaching a crisis point (sometimes despite many attempts at help-seeking) before they were taken seriously, or before appropriate help was offered to them. They were frustrated and wanted to earlier access points which would assist with longer term engagement with services and intervention:

“I just think it's sad that you have to have something super severe for them to take you seriously.” (Youth #6)

##### 3.2.2.4. Reduced stigma

Would facilitate help-seeking. Participants described that the “stigma against mental illness is terrible” (Family Member) and that work needs to be done about educating youth, service providers, and others who interact with youth around mental illness to reduce the stigma:

“I hear them [youth with psychosis] say they don't want to be seen as crazy….unfortunately, that is the stigma out there.” (Service Provider #4)

##### 3.2.2.5. EPI approach

Families and youth described the benefits of the EPI programs in meeting the needs of youth with psychosis. They especially noted important features including, intensive case management (i.e., “assertive outreach”), psychoeducation for youth and their families, a trusting and engaging relationship with an case manager, support from the acute phase of the illness, a psychiatry component and a focus on recovery. The team-based service delivery approaches was also noted because intervention for psychosis can not be achieved with a solo practitioner. Multi-disciplinary teams that included “nurses, family workers, recovery care, psychiatry, psychology” were said to be beneficial in that it allows for holistic and family or youth-focused care:

“I’m just pleased with the team approach to it. Like, it's good that there is a team … I feel it is good there is a team of people looking after him because … so, if he calls in and one person is not there, he can talk to another person. (Short pause.) So, overall, it's been pretty positive.” (Family Member #8)

### 3.3. Knowledge translation workshop

**Figure 5:**
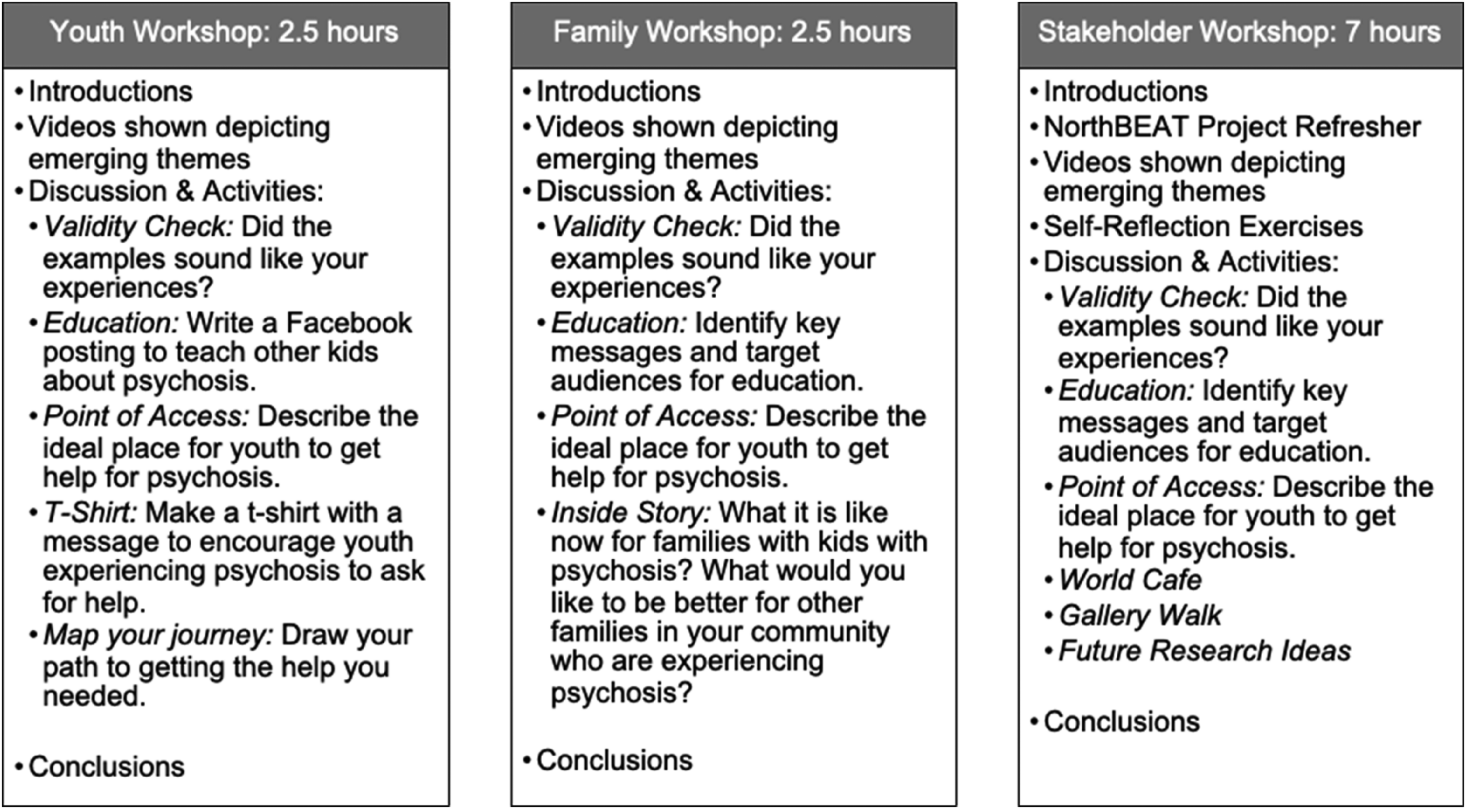
Knowledge translation workshop agendas.

The youth and family participants who were part of early psychosis intervention services and consented to be contacted after their interviews were invited to the youth and family workshops and travel costs were covered (agendas found in Figure 5). Participation rates were 30% of invited youth and 83% of the invited family participants.

For the stakeholder workshop, invitations were sent to all service provider participants who consented and were still in their role in the services. Additional invitations were sent to program managers at each of the project sites, program decision makers or funders, all of the advisory group members and key Indigenous community partners. 70% of the invited stakeholders attended the stakeholder workshop.

As outlined in the methods section, youth, family, and stakeholder workshops were held separately. Each workshop began with videos which provided an overview of the emerging themes in the data. Immediately after the viewings, the workshop facilitator conducted a validity check to determine how closely the presented data matched the experiences the participants were intending to share.

While the presented data were largely found to be congruent with what the participants had intended, some conflicting perspectives emerged between participants. Some non-Indigenous participants described that for Indigenous youth, treatment delays may be associated with a perception that hallucinations (a hallmark sign of psychosis) are a “gift”. However, at the data validation workshop, participants and stakeholders provided other perspectives. Another example provided by Indigenous leaders also explained that while children are closest to the Creator, and it is culturally accepted in some communities that children can communicate with the Creator by hearing or seeing things (that perhaps others do not experience), the line is crossed when these experiences result in self harm, suicidality or harm to others. In cases where these experiences lead to someone getting hurt, this is not the same cultural or spiritual belief:

“I think it's really important that we increase the public awareness there so that we can try to have some discussions around what is the difference between some of these spiritual or cultural experiences and psychosis”. ….” I think spiritually and culturally, you know, having visions or listening to deceased Elders would be spiritually uplifting. Where symptoms of psychosis, generally are not uplifting”. Indigenous Service Provider

**Figure 6:**
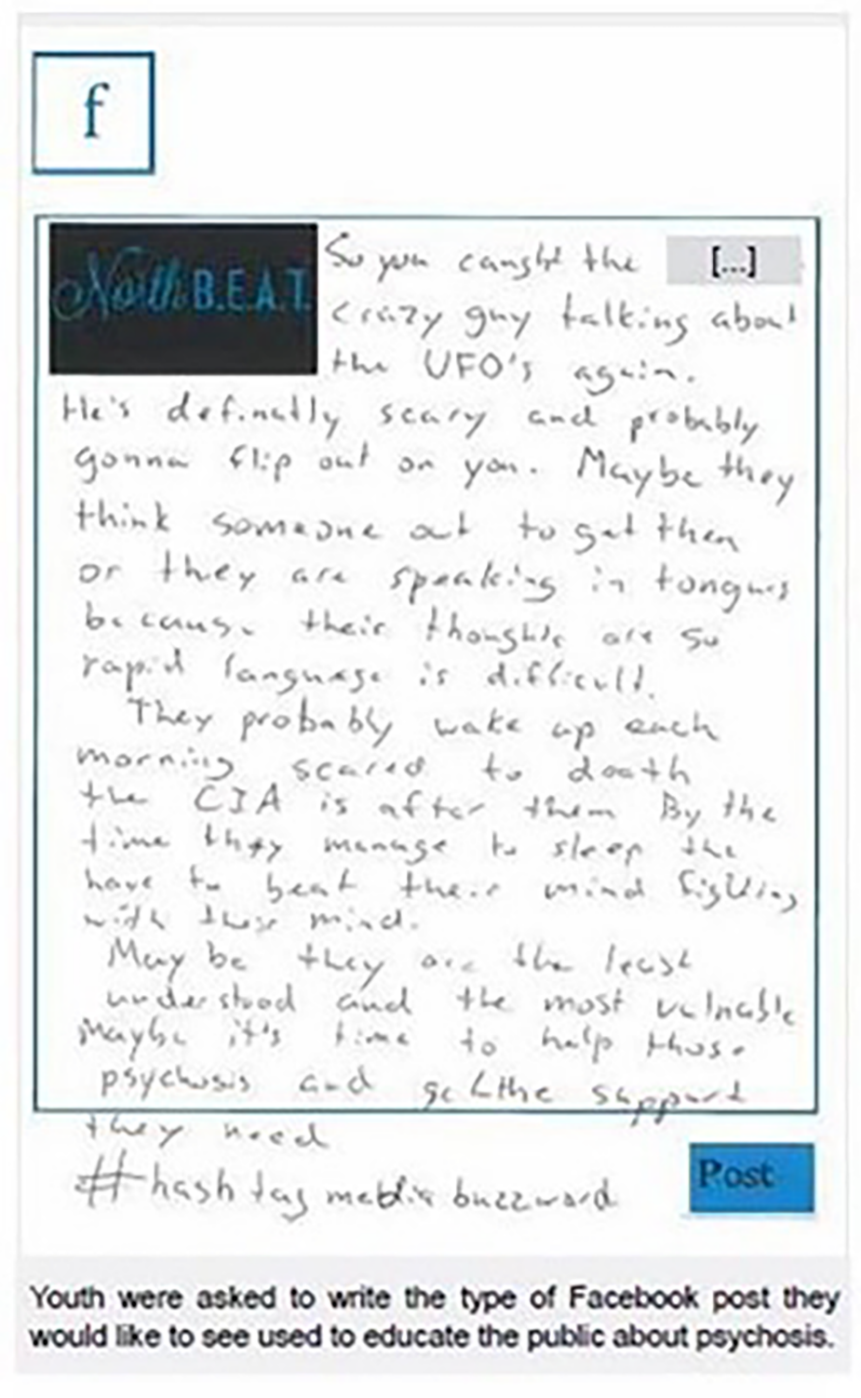
Facebook posting created by youth participant.

After the data validation, each workshop had a participant-guided educational activity. Youth completed an exercise where they wrote a sample Facebook post that educated other kids about psychosis (Figure 6). Families and stakeholders identified key educational messages regarding psychosis and target audiences. The need for increased communication between different sectors and the public was identified as a major issue. Potential focus for increased education included: social and community services, children's services, educators, primary and acute healthcare, police services, emergency medical services, and youth and families.

**Figure 7:**
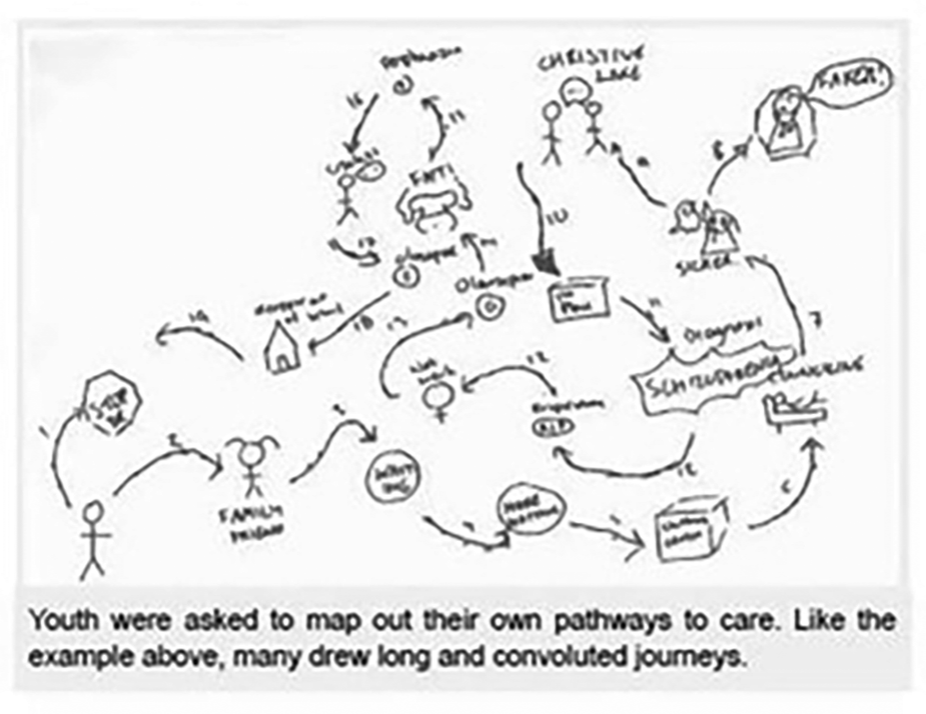
Pathway to care created by youth participant.

In the “point of access” exercise, youth, families, and service providers described the ideal place for youth to access help for psychosis. In addition to this, youth completed a mapping exercise where they visually depicted their journey to accessing EPI services (Figure 7, lower left corner). Stakeholders expressed surprise at the convoluted pathways these youth had to take as a result of the lack of awareness amongst health care professionals:

“Seeing their paths made me feel sad and angry.” “…Young people and their families are often not believed.”

**Figure 8:**
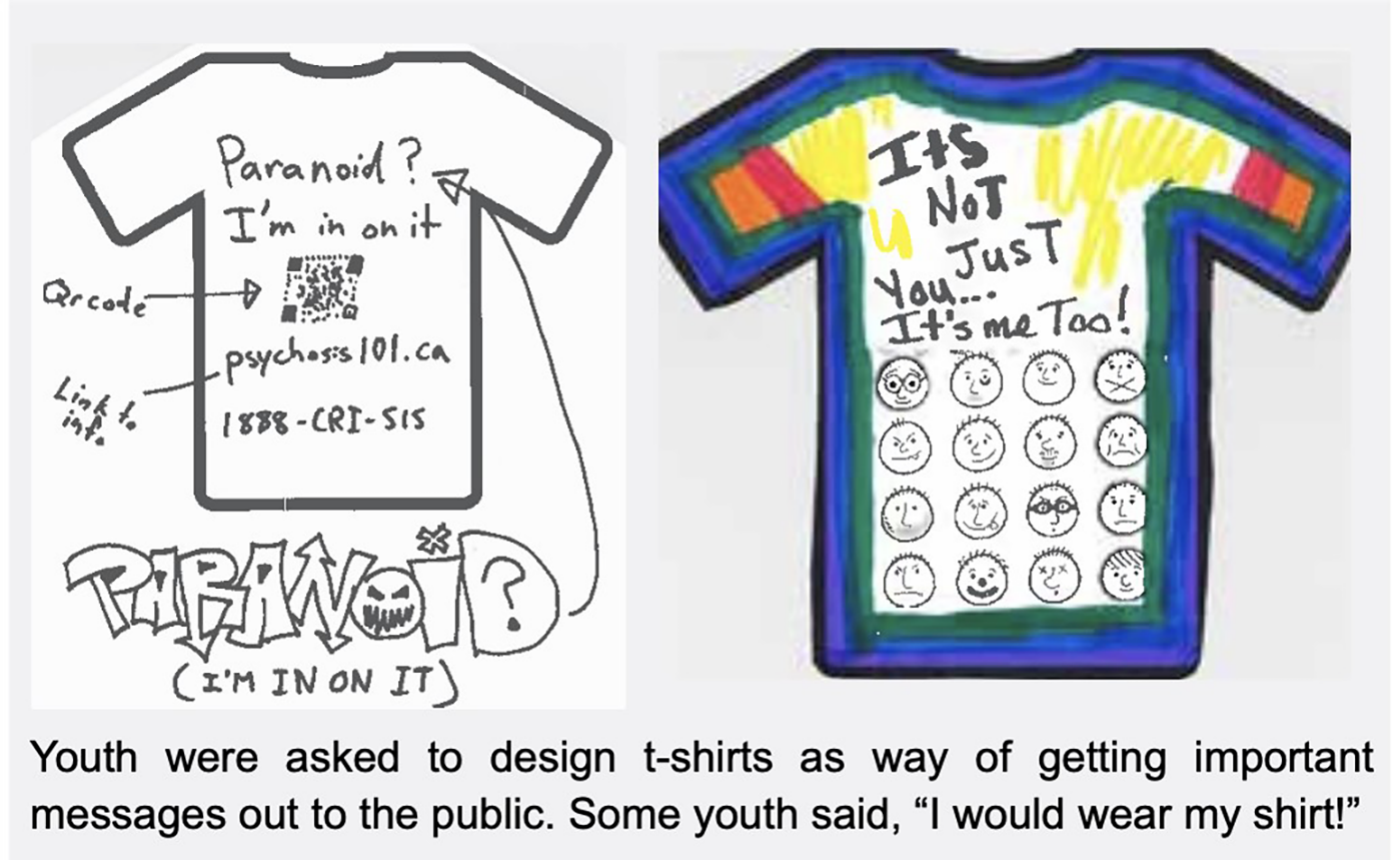
Youth-created art products.

As seen in Figure 8, youth participated in arts-based activities which reflected their experiences living with psychosis. They created t-shirts which encouraged other youth experiencing psychosis to seek help. Stakeholders had the opportunity to do a “gallery walk” of the art products youth and families had created. Their personal reflections included:

“How do we as healthcare providers give more resources/teach parents/families/partners to be ‘there’ for their child/adolescent/partner?”

“Continued need for acceptance of illness to not isolate people and stigmatize”

“There is still need to improve: more education; to reduce stigma; increase capacity; access services quickly; families continue to need so much support during and after child has suffered from psychosis”

Families shared stories about living with psychosis, and provided insights into what would be beneficial for others and their communities. The need to travel long distances to access treatment, an isolated and disconnected system, and lack of education were identified as barriers. Suggested future projects to address these needs included: developing and testing models of interaction between primary care providers and specialist services, improving the referral process between children and adult mental health, coordinating services between different government programs, and addressing academic barriers during the first episode. Participants suggested that cross-sector collaboration was needed to mitigate the barriers to early assessment and treatment which were identified in this study.

## 4. Discussion

The NorthBEAT research project sought to understand the perceived service needs of Indigenous and non-Indigenous youth in Northern Ontario who experience first episode psychosis. The structured scales indicate that in our sample of youth, their mental health status was functionally better than expected. For example, 90% of them were coded as 60 or higher (i.e., mild to moderate symptoms) on the GAF (Global Assessment of Functioning Scale). Mean scores on the PANSS (Positive and Negative Symptom Scale) indices, suggested they were experiencing less symptoms and general psychopathology compared to the normative sample (medicated patients with schizophrenia). Half of the participants (*n* = 13) had clinically significant BSI (Brief Symptom Inventory) scores. They also had generally a more positive outlook on their recovery; they generally had high levels of hope and confidence and success orientation, and were not dominated by psychosis symptoms. On the other hand, there was a wide range in duration of untreated psychosis (DUP) from 1 to 96 months, with only 23% having a DUP under the recommended 3 months.

Youth in Northern Ontario face similar barriers to receiving early intervention as their urban counterparts, such as lack of knowledge about psychosis and psychosis services. They share many of the needs perceived by urban youth, including greater public psychosis awareness and education, reduced stigma, shorter wait times, and EPI specific model of care (43). However, the results from this study identified additional, significant barriers to EPI and service needs that are unique to both Indigenous and non-Indigenous youth living in northern communities. Youth living in northern communities face further barriers specific to their geographic context. The resources in the community may be few and restricted by age, or geographic area and catchment/boundaries. Travelling long distances (often hours by car) may not be a feasible solution for a number of reasons including poverty, lack of vehicle or other social determinants of health.

Indigenous youth and their families face additional barriers. The consequences of colonialization, residential schools and systemic racism has led to mistrust of healthcare providers, including those providing early intervention in psychosis. As a result, one strategy for coping with racism in healthcare settings is the avoidance of hospitals and nursing stations (44). Limited cultural competency and sensitivity training for non-Indigenous healthcare providers impedes their ability to engage in productive, respectful dialogue with Indigenous patients (45). Even in this project, we found different rationales from Indigenous and non-Indigenous participants regarding the reasons for delayed treatment; a non-Indigenous service provider wondered if the delay was due to hallucinations being more accepted in some Indigenous communities. Whereas this belief was challenged during the data validation workshop, Indigenous stakeholders explained that hallucinations are not more culturally accepted, and the delays are from systemic barriers that Indigenous communities experience.

Further, the participants in this study spoke about experiencing *double stigma*, the stigma of experiencing mental illness, and the stigma of being Indigenous in the healthcare system. In Canada, the discovery of unmarked graves at historical residential school sites, and Indigenous people dying from preventable deaths due to systemic racism seem to support what the participants in this study have shared. Indigenous, Inuit and Metis youth and their families face additional systemic barriers in their journey to seek early intervention.

A possible explanation for youth participants functioning relatively well is the limited number of structural interviews conducted. Despite many attempts to increase these numbers, the total sample size for the structured interviews remained low. Perhaps the sample was skewed to the healthier and more functionally well youth who agreed to participate in the structured interviews. Recruitment sites and youth participants suggested two reasons behind recruitment difficulties; (i) eligible youth were reluctant to participate; and (ii) program changes in human resources which led to a smaller eligibility pool than anticipated. The recruitment sites explained that some of their eligible youth were not interested in participating because they were in recovery, and did not want to revisit the time in their life when they were most ill (e.g., they just “want to move on with life”). Additionally, between development of project protocol and recruitment some of the project sites underwent restructuring to deal with resource constraints. This resulted in them having to increase the age of eligibility for their services, and ultimately a fewer number of clients under 18 years. Though disappointing in terms of recruitment, this challenge in recruitment illustrates one of the subthemes revealed through our narrative interviews, that services with limited resources must enact wider age ranges for eligibility for their services. This may be a reflection of the challenges youth with psychosis face in accessing services.

While this study identified some of the barriers that rural youth face in seeking care, further research is needed to examine how to design and implement educational and clinical interventions which are tailored to local contexts. In the time since the original study, the proliferation of telehealth presents an opportunity to investigate how these technologies can be used to overcome geographic challenges in delivering EPI to rural and remote areas.

## 5. Conclusion

NorthBEAT sought to understand the service needs of youth and their families experiencing first episode psychosis in Northern Ontario, Canada. Findings from this study suggested that although Northern Ontario youth (particularly Indigenous youth) experiencing psychosis share similar needs as urban youth, they face additional challenges as a result of their unique geographical and cultural contexts. Though based in a northern Canadian rural context, it is hoped that these findings are helpful to services in other rural regions as well. From these findings, we present practice and policy implications that will be useful for EPI services that aim to provide equity, quality care to remote, northern populations.

## 6. Policy implications

The NorthBEAT research project represents initial steps towards understanding how to decrease the barriers to early assessment and treatment, and meet the service needs of youth who experience psychosis in northern Ontario. Now that we have a greater understanding of what the needs and barriers are, we have an opportunity to do better.

### 6.1. Consider youth friendliness in service mandates and eligibility criteria

Youth friendly and family friendly services are the hallmark of EPI services (9). This includes flexibility with service mandates and eligibility criteria. When eligibility criteria are rigid, and narrowly applied, the barriers are accentuated, especially in rural and remote regions. Youth or their families seeking help find navigating the mental health care system convoluted. Rigid service criteria serves to isolate them further.

### 6.2. Increase awareness about psychosis and how to intervene

People who work with youth need to be able to recognize youth struggling with psychosis and be aware how to connect them to services that can intervene. This awareness needs to go beyond social service and health care providers; awareness needs to reach the teachers, coaches, and first responders that may be the first contact with youth. This is consistent with the public awareness campaigns among EPI services (46). Frontline providers need more education about early psychosis, clinical training to assess signs of distress, and sensitivity training for handling conversations with youth who are seeking help.

### 6.3. Improved collaboration across sectors

There needs to be better synchronicity and collaboration amongst funders (e.g., across provincial Ministries) and sectors who interact with youth. This collaboration is necessary to create the reality “every door is the right door”. Aside from healthcare, other Ministries including those responsible for athletics, education, social services, justice and child welfare need to be part of the conversations. Youth intersect with all these sectors.

### 6.4. Address unique challenges to people living in rural and remote geography

People living in rural, remote and northern communities have to travel long distances at times to receive health services. Furthermore, the lack of specialized services or staffing increases the delay, and may worsen outcomes. Adequate funds for travel, coupled with creative multi-modal solutions such as telehealth will facilitate early intervention.

### 6.5. Specifically address barriers to indigenous youth and families

Given the historical and systemic barriers faced by many Indigenous communities, we have an opportunity to do better by intentionally addressing the barriers. For example, education about cultural competency and humility may help to address the double stigma Indigenous youth with mental health difficulties face accessing services. Over-generalization across all Indigenous people is also a barrier. With truth and reconciliation in mind, we need to be mindful of our biases, and address the socioeconomic barriers that face Indigenous youth.

## Data Availability

The raw data supporting the conclusions of this article may be made available by the authors without undue reservation until December 31 2026, at which point the data will not be available because as per local research ethics policy the raw data will be safely destroyed. Further inquiries can be directed to the corresponding author.
